# Angular kinematics during top speed sprinting in male intercollegiate track and field and team sport athletes

**DOI:** 10.3389/fspor.2025.1535798

**Published:** 2025-03-31

**Authors:** Kenneth P. Clark, Christopher R. Meng, Cory T. Walts, Laurence J. Ryan, David J. Stearne

**Affiliations:** ^1^ Department of Kinesiology, West Chester University, West Chester, PA, United States; ^2^ Department of Athletics, Princeton University, Princeton, NJ, United States; ^3^ Department of Athletics, University of Pennsylvania, Philadelphia, PA, United States; ^4^ Independent Researcher, Dallas, TX, United States

**Keywords:** running biomechanics, top speed sprinting, locomotor performance, track and field, team sports

## Abstract

In this investigation we examined lower extremity angular kinematics and top speed sprinting performance in 98 male intercollegiate athletes with backgrounds in either track and field (TF, *n* = 28) or team sports (TS, *n* = 70). Athletes completed 40 m running trials, with high-speed video recorded from 30–40 m, and 2D sagittal plane motion analysis. Key kinematic variables included: maximum thigh extension and flexion during the swing phase, leg and foot angles of the stance leg at touchdown, swing-leg thigh and knee angles at contralateral touchdown, leg excursion angle during the ground contact phase, thigh total range of motion during the swing phase, and thigh angular velocity and acceleration. Our first hypothesis was that each key kinematic variable would be significantly correlated with top speed both across the entire sample of participants and within groups of TF and TS athletes. Our second hypothesis was that sub-groups of TF and TS athletes of similar top speeds would demonstrate significantly different angular positional strategies. The first hypothesis was partially supported, as each key kinematic variable was significantly correlated with top speed when analyzed across the entire heterogeneous sample (0.30 ≤ |*r* or *ρ*| ≤ 0.66, *p* < 0.05), but most were not significantly correlated when analyzed within groups of TF or TS athletes. The second hypothesis was fully supported, as substantially different angular positions were demonstrated by Slow TF and Fast TS athletes of similar top speeds, with Fast TS athletes typically exhibiting a less front-side and more ground-based strategy compared to their Slow TF counterparts. In contrast to the angular position variables, the physical capacity to rotate the limbs (thigh angular velocity and acceleration) was correlated with top speed both across the entire sample of participants and within groups of TF and TS athletes. Therefore, this study indicates that when coaching and training team sport athletes, more specific kinematic models may be beneficial for technique and performance enhancement during top-speed sprinting.

## Introduction

Top speed sprinting performance is crucial for athletes in a variety of sports, both in track and field (TF) and team sports (TS) (1, 2). With respect to kinetic determinants of performance, faster top speeds are linked to greater rates of vertical force application, greater net propulsive force during transitional acceleration, and increased vertical stiffness (3–6). For spatiotemporal kinematics, determinants of top speed include shorter ground contact times, faster step rates, and longer step lengths (5, 7, 8). In addition to the kinetic and spatiotemporal variables, it is also important to explore the angular kinematics that are related to top speed performance.

Prior research has linked several angular kinematic parameters to better sprinting performance. First, faster top speeds typically correspond with a more “front-side” kinematic strategy, highlighted by decreased peak thigh extension behind the body and increased peak thigh flexion in front of the body during the swing phase (9). Second, the positioning of the touchdown leg has been noted as a critical factor, with faster runners usually exhibiting a smaller angle from the center of mass (COM) to the foot and a forefoot ground contact pattern (10, 11). Third, the orientation of the swing leg at contralateral touchdown may also be important, as a more forward position of the swing thigh and a more flexed angle of the swing knee at contralateral touchdown have been recommended (12). Fourth, at top speed faster runners tend to spend a larger percentage of the gait cycle in the air and a smaller percentage of the gait cycle on the ground (3, 4), and this may be kinematically reflected in a larger thigh total range of motion during the swing phase and a smaller leg excursion angle during the ground contact phase (7). Finally, the capacity to generate rotational limb speed is important, as magnitudes of thigh angular velocity and thigh angular acceleration have been correlated with top speed (7, 13–15).

Although the aforementioned variables are strongly correlated with top speed in heterogeneous samples, statistical relationships may become less prominent when examined in relatively uniform groups of athletes (2, 16) and additional research is needed to clarify how tightly each variable couples with top speed in homogeneous groups of TF or TS athletes. Furthermore, although technical models of sprinting are well established for TF athletes (9), at present there have been few studies directly comparing the angular kinematic strategies employed by TF vs. TS athletes during top speed sprinting. Traditional TF technical models for sprinting may not be optimal for TS athletes given the typical constraints of team sport game play (17). Recent research suggests that TF and TS athletes might utilize different mechanics even when running at similar speeds, with TS athletes clearly demonstrating a more “ground-based” strategy compared to their TF counterparts. TS athletes may invoke this strategy for a variety of reasons, including the reactive agility and repeated sprint demands of gameplay, or holding a ball or implement while running. This ground-based strategy includes longer ground contact times and contact lengths, shorter flight times and flight lengths, and increased duty factor (18). However, the corresponding manner in which these spatiotemporal variables are attained by the angular kinematics and positioning of the lower extremity remains to be elucidated and requires further investigation.

Therefore, in this investigation we examined lower extremity angular kinematics during top speed sprinting in male intercollegiate athletes from a variety of sporting backgrounds (TF and TS athletes). Based on prior experimental research and applied coaching experience, our first hypothesis was that each of the following key kinematic variables would be significantly correlated with top speed: maximum thigh extension and flexion during the swing phase, COM-foot angle of the stance leg at touchdown, foot angle of the stance leg at touchdown, swing-leg thigh and knee angles at contralateral touchdown, leg excursion angle during the ground contact phase, thigh total range of motion during the swing phase, and average thigh angular velocity and peak thigh angular acceleration during the entire gait cycle. We expected that each variable would be correlated with top speed both across the entire sample of participants and within groups of TF and TS athletes. Our second hypothesis was that when analyzing sub-groups of TF and TS athletes of equivalent top speeds, different angular positional strategies would be utilized to attain similar top speeds. Specifically, compared to TF athletes of similar speed, we expected that TS athletes would demonstrate a positional strategy that was less front-side (e.g., increased maximum thigh extension and decreased maximum thigh flexion during the swing phase), more ground-based (e.g., larger leg excursion angles during the ground contact phase and decreased thigh total range of motion during the swing phase), and with more extended angular positions at touchdown (stance-leg angle, swing-leg angles of thigh and knee).

## Methods

### Participants

The experimental protocol (18) was conducted at three universities with male varsity intercollegiate student-athletes (*n* = 98) actively participating in track and field (*n* = 28) and team sports (*n* = 70). The participants provided written informed consent in accordance with the Institutional Review Board for each university which had approved the study (WCU: IRB# FY2022-361; University of Pennsylvania: IRB# 852005; Princeton University: IRB# 15345). The study was conducted in accordance with the Declaration of Helsinki.

The 98 male participants [mean ± standard deviation (SD), age: 19.60 ± 1.35 years, height: 1.80 ± 0.06 m, mass: 81.17 ± 8.82 kg, body mass index: 24.93 ± 2.37 kg/m^2^] had passed a medical exam prior to joining their team for the season and were injury-free during the testing period. The group of 28 track and field athletes (18.79 ± 1.17 years, height: 1.79 ± 0.05 m, mass: 75.13 ± 6.14 kg, body mass index: 23.59 ± 2.12 kg/m^2^) participated in sprints (≤400 m), horizontal jumps, and/or hurdle events. The group of 70 team sport athletes (19.93 ± 1.28 years, height: 1.81 ± 0.07 m, mass: 83.59 ± 8.59 kg, body mass index: 25.47 ± 2.26 kg/m^2^) participated in lacrosse (*n* = 41), soccer (*n* = 18), and baseball (*n* = 11).

### Experimental protocol

Each participant performed 40 m sprint tests at their respective university track facility. An indoor track facility was used at one university and outdoor track facilities were used at the other two universities. Each university had a standard rubberized running track. Test sessions were performed on the outdoor tracks during dry and temperate weather conditions with minimal wind.

After receiving signed consent forms, the height and weight of the participants were recorded. Participants wore their own preferred athletic clothing and running sneakers (no spikes, cleats, or studs). Participants completed their individual warm-up routines as prescribed by their coaches. For all participants, warm-up activities included jogging, dynamic stretches, plyometric-type exercises (e.g., skipping activities), and sub-maximal sprints. The participants then completed two 40 m sprint trials starting on their own initiative from an upright two-point stance. Participants were instructed to perform maximum-effort acceleration and maintain maximum speed through the 40 m finish-line. A minimum of four minutes of rest between trials was provided to allow for complete recovery.

Video and timing data were recorded from 30–40 m for each trial where the athletes attained maximum or near-maximum velocity (19). Video was recorded at 240 frames/s using a high-speed, high-resolution camera (Apple iPhone, Apple USA, Cupertino, CA) mounted to a tripod at a height of 1.0 m and placed 10 m perpendicular to the running lane centered at 35 m. The corresponding split time was measured using an automatic timing system (Speed Trap II, Brower Timing Systems, Draper, UT). Average running speed for the 30–40 m interval was determined and the faster sprint trial from each participant was used for the video analyses.

### Video analyses

Kinovea (v. 0.9.5, GPL v2 license) was used to digitize each video. The digitized pixel positional data were then exported to Microsoft Excel for 2D kinematic analyses. Spatial calibration of each video was performed by digitizing the running lane boundaries of the 30–40 m field of view at the near and far lane lines at 30 m and 40 m to create a pixels-per-meter conversion factor. A full stride cycle consisting of one complete step from each leg that occurred in the most central field of view near 35 m was analyzed. This selection was made for highest accuracy and to minimize perspective error in the video analysis (20).

**Figure 1:**
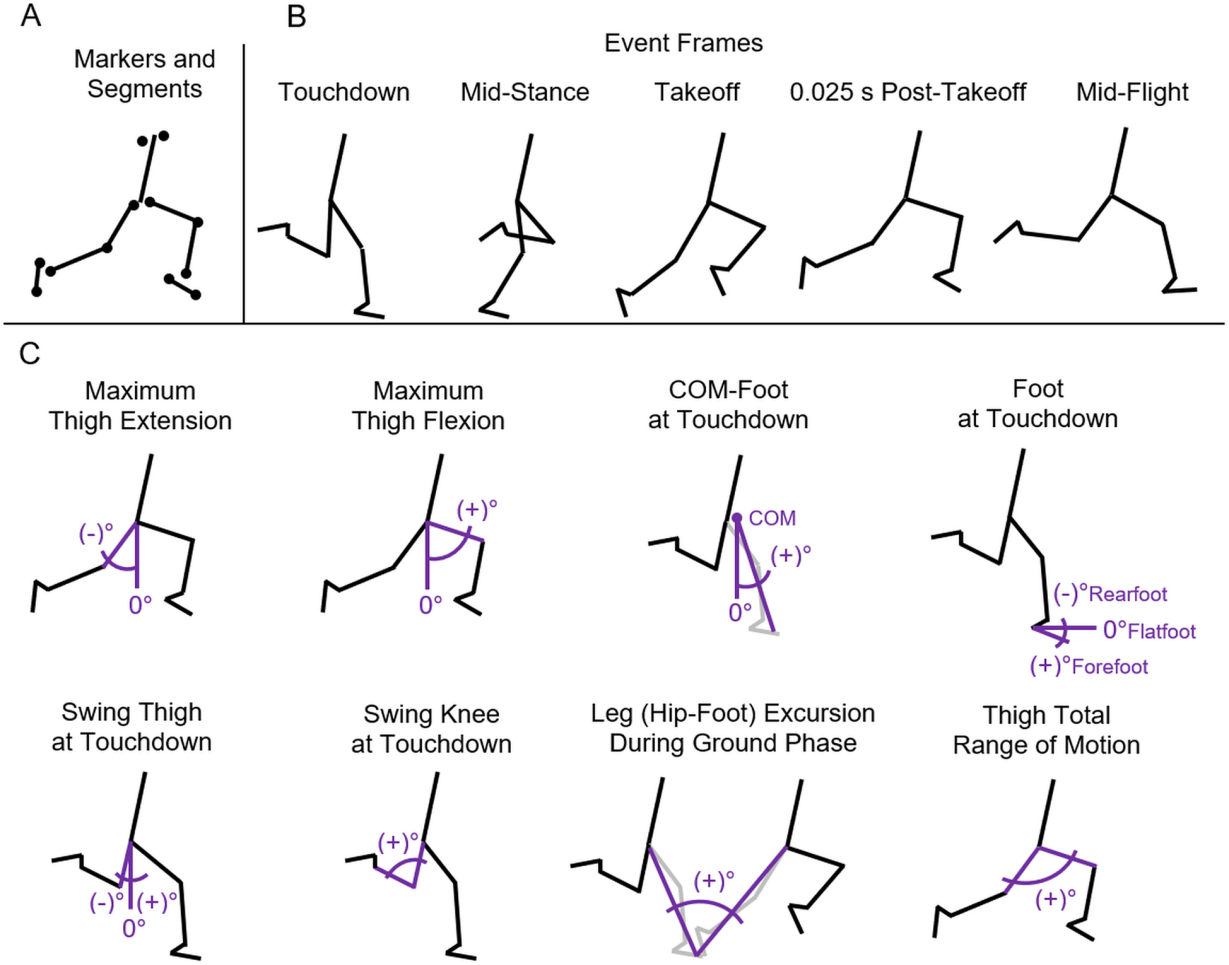
Kinematic definitions. **(A)** Twelve marker locations were digitized at the toes and heels of the shoes, and the joint centers of rotation of the ankles, knees, hips, and shoulders to create a seven-segment model. **(B)** Five event frames for each step were digitized. **(C)** Conventions for the key angular position variables.

Five event frames for each step were selected for the analyses (Figures 1A,B). Thus, the following 11 discrete frames during the full stride cycle (left and right steps) were digitized: first touchdown, mid-contact, first takeoff, 0.025s post-takeoff, mid-flight, second touchdown, mid-contact, second takeoff, 0.025s post-takeoff, mid-flight, and third touchdown. For each ground contact phase, the frames of touchdown and takeoff were defined as the first and last frames, respectively, where the running shoe was clearly in contact with the ground. The frame at 0.025s post-takeoff occurred six frames after the previous takeoff frame, based on the video frame rate of 240 frames/s. The frame at mid-flight was calculated from the midpoint frame of the touchdown and takeoff frames. This frame selection strategy accurately captured the ground contact phase and the thigh segment total angular range of motion where front leg peak thigh flexion and rear leg peak thigh extension occur (21).

Specific body landmarks were digitized to create markers which established their positions in the sagittal plane at each of the 11 frames of the stride cycle (Figure 1A). A total of 12 markers, six on each side of the body, were used to quantify body segment positions at each frame. Landmarks were digitized at the following locations: the most inferior and posterior point on the shoe (heel), the most inferior and anterior point on the shoe (toe), the lateral or medial malleolus (ankle), the center of rotation of the knee joint, the center of rotation of the hip joint, and the center of the rotation of the shoulder joint. Positional data from the 12 markers were then used to form a seven-segment model which consisted of the foot segments, shank segments, thigh segments, head-arms-trunk segment, and the corresponding COM (22). Additional reference lines from the COM location to the foot segment and from the hip marker to the foot segment were generated. All videos were digitized by the same investigator (second author). A subset of ten videos was digitized twice with at least several weeks between analyses to evaluate intra-rater reliability for the key kinematic variables (see Results).

### Key kinematic variables

Basic spatiotemporal variables from this data set have been previously reported (18), including: contact time, flight time, step rate, step length, contact length, flight length, and duty factor. Running speed (*Speed* = *SL* • *SR*) was determined from the product of step length and step rate during the analyzed stride cycle. For the present investigation, the values for the key kinematic variables were determined from the marker positions and seven-segment model at each frame of analysis. The key angular position variables included maximum thigh extension angle, maximum thigh flexion angle, COM-foot angle at touchdown, foot angle at touchdown, swing thigh angle at contralateral touchdown, swing knee angle at contralateral touchdown, leg excursion angle (hip-to-foot line segment incorporating the thigh, shank, and foot angles) during the ground contact phase, and thigh total range of motion (Figure 1C). Key angular rate variables for thigh motion were also calculated. Average thigh angular velocity (*ω_avg_* = *θ_rom_*/*t_step_*) was determined from the thigh range of motion during the time for each step (7). Peak thigh angular acceleration (*α_peak_* = 2π^2^ *θ_rom_ f* ^2^*_stride_*) was determined from the thigh range of motion and the stride frequency (13). The derivation of this equation is based on the sinusoidal motion of the thigh. For the top speed trials of the 40 participants in that study (13), a pure sine wave function was fit to the angular data from each thigh with a mean goodness of fit *R*^2^ = 0.964 ± 0.012 and a total range of *R*^2^ = 0.934–0.984 for all 80 waveforms. Since the angular motion closely follows a sine wave, peak thigh angular acceleration can be calculated from the *α_peak_* equation instead of using numerical computations involving low-pass filters and second derivative signal processing routines of time series data of thigh position (13). To link the joint angular kinematics during the ground contact phase with kinetic variables, measures of force and stiffness were estimated from the spatiotemporal values (see Supplementary Materials). All values reported for each of the key variables were average quantities from the left and right steps over one complete stride cycle.

### Statistical analysis

To test the first hypothesis, correlational analysis was performed to determine the relationship between top speed and each of the key angular position and rate variables. For each variable, the normality of data was checked using the Shapiro-Wilk test. Normally distributed data were analyzed with Pearson's *r* and non-normally distributed data were analyzed with Spearman's *ρ*. For graphic purposes, linear regression was also performed to generate a best-fit equation with variable *x* representing top speed. The correlational analyses and linear regression were completed across the entire sample and also separately for the groups of TF and TS athletes.

To facilitate interpretation of the data, participants were categorized into five sub-groups: Fast TF [top speed > 9.65 m/s (*n* = 14)], Slow TF [top speed < 9.65 m/s (*n* = 14)], Fast TS [top speed > 9.00 m/s (*n* = 22)], Intermediate TS [top speed 8.50–9.00 m/s (*n* = 29)], and Slow TS [top speed < 8.50 m/s (*n* = 19)]. For the experimental sprint variables, mean ± SD values were calculated for each sub-group.

To test the second hypothesis, sub-groups of “Slow TF” and “Fast TS” athletes were evaluated to examine differences in running mechanics for athletes with comparable top speeds. This included 14 Slow TF athletes and 22 Fast TS athletes with similar mean top speed for each sub-group (Slow TF: 9.34 ± 0.21 m/s; Fast TS: 9.31 ± 0.19 m/s). After confirming the normality of data using the Shapiro-Wilk test, separate independent *t*-tests were conducted for each variable to specifically examine significant differences between the sub-groups of Slow TF and Fast TS athletes. Absolute and percentage differences between the two sub-groups were also quantified, with percentage difference computed as: (|*Slow TF*—*Fast TS*|)/[(*Slow TF* + *Fast TS*)/2] ⦁ 100.

Additionally, the Supplementary Materials contain segment angles for the five sub-groups, with correlational analyses for the lower extremity joint angles at each of the five event frames. For all statistical tests, the *a priori* threshold for significance was set at *α* = 0.05. For the key kinematic variables, multiple comparisons were not controlled for because only a limited number of *a priori*, scientifically logical comparisons were analyzed as part of the original experimental design (23–25). All statistics were completed using Microsoft Excel and GraphPad Prism software (version 9, San Diego, CA).

## Results

The fastest trial for each of the 28 TF and 70 TS athletes were analyzed, for a total of 98 videos. The average top speed for the TF group was 9.67 ± 0.39 m/s (range: 8.96 to 10.35 m/s), including seven athletes with a top speed greater than 10.0 m/s. The average top speed for the TS group was 8.79 ± 0.46 m/s (range: 7.61 to 9.70 m/s). For the intra-rater reliability tests, the mean absolute percent error for the key kinematic variables was 1.73 ± 0.73% between the first and second intra-rater analyses.

**Figure 2:**
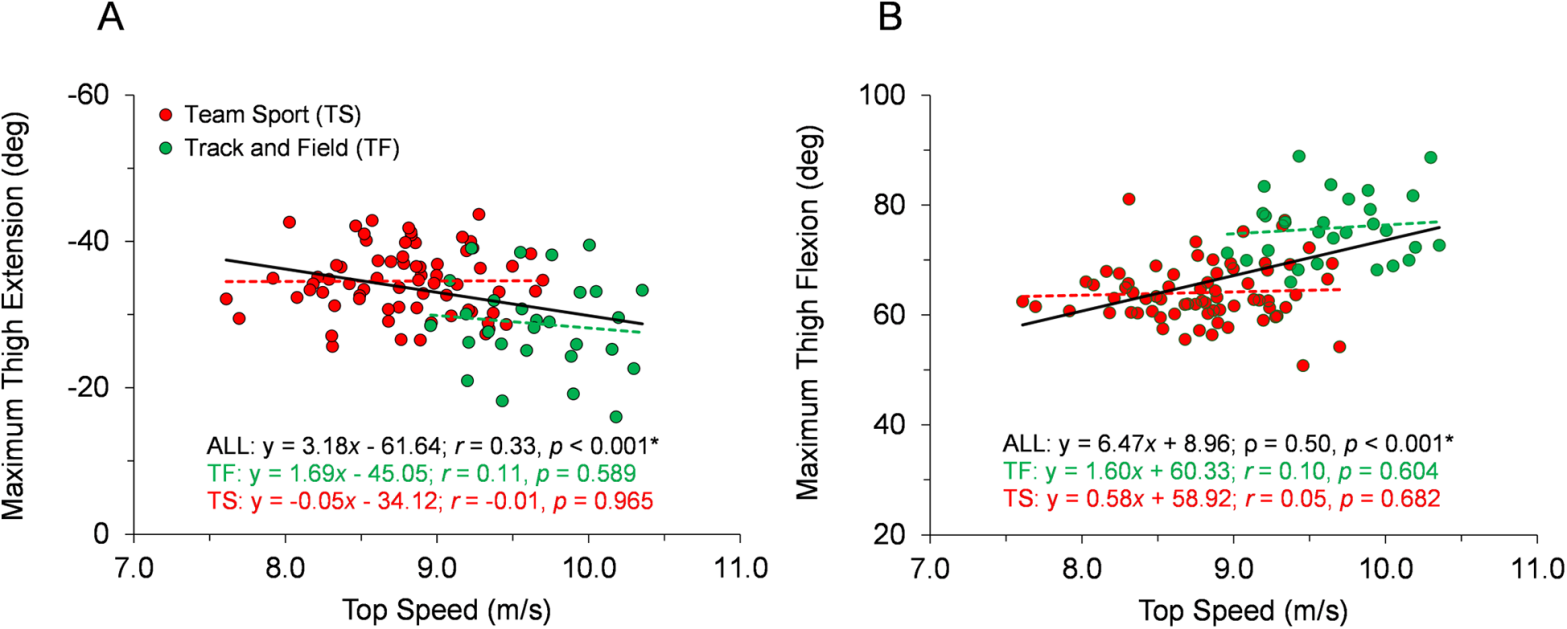
Angular position data across the range of top speeds. Trend lines with best-fit linear regression equations, correlation coefficients (Pearson's *r* or Spearman's *ρ*), and *p*-values (* indicates significant) are shown for the entire sample and for each group of TF and TS. **(A)** Maximum thigh extension angle. **(B)** Maximum thigh flexion angle.

**Figure 3:**
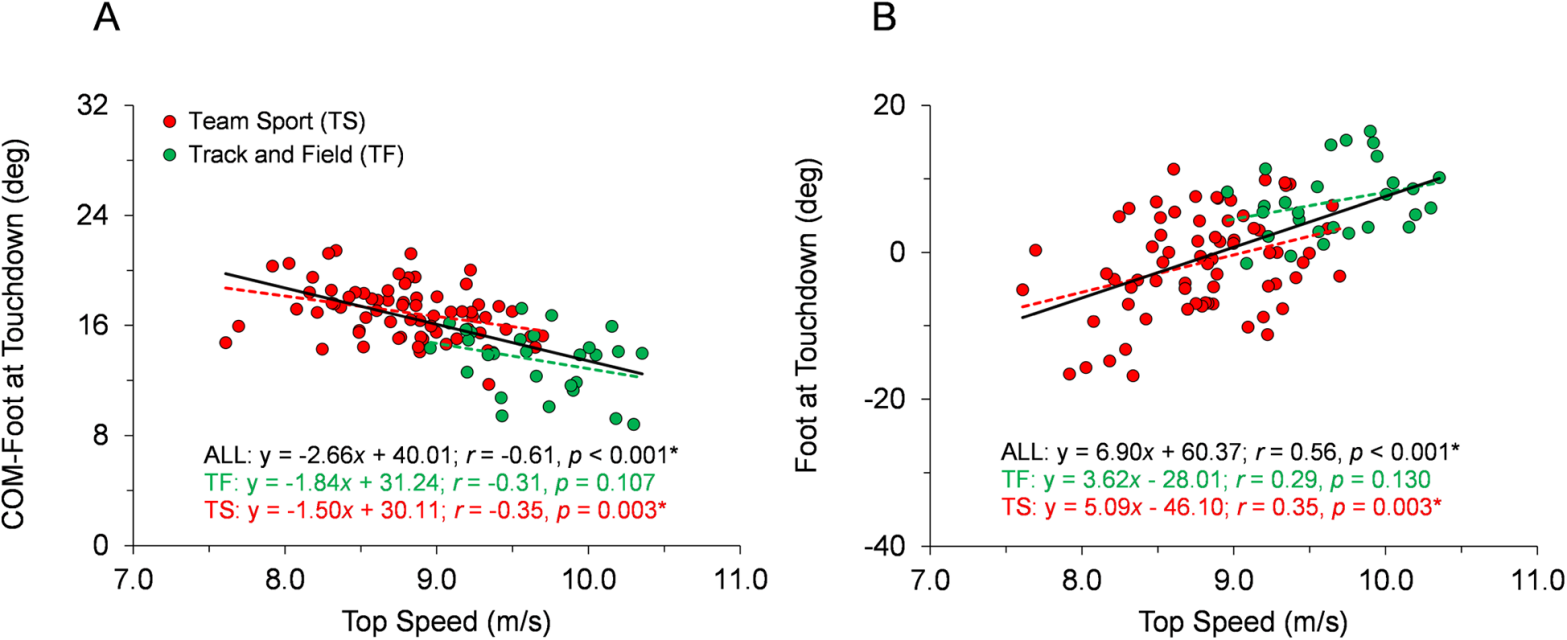
Angular position data across the range of top speeds. Trend lines with best-fit linear regression equations, correlation coefficients (Pearson's *r* or Spearman's *ρ*), and *p*-values (* indicates significant) are shown for the entire sample and for each group of TF and TS. **(A)** COM-foot angle at touchdown. **(B)** Foot angle at touchdown.

**Figure 4:**
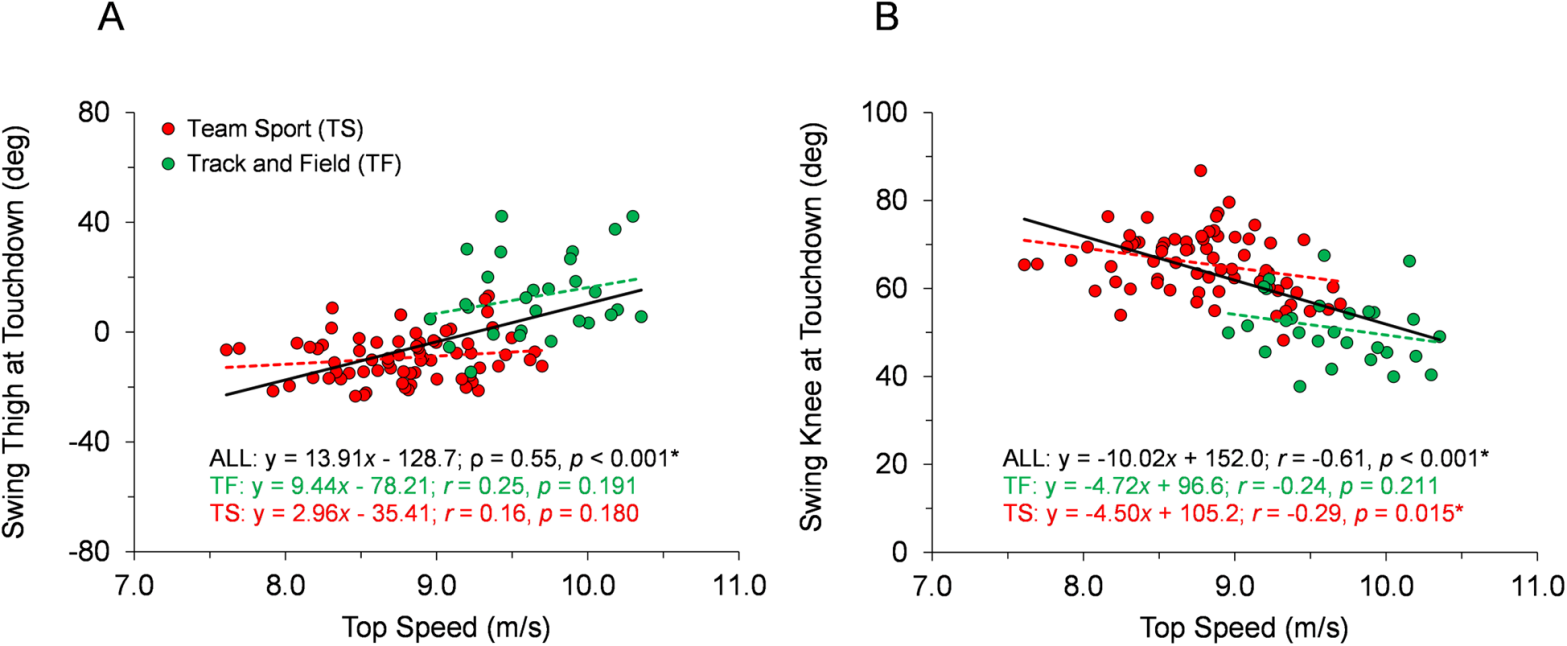
Angular position data across the range of top speeds. Trend lines with best-fit linear regression equations, correlation coefficients (Pearson's *r* or Spearman's *ρ*), and *p*-values (* indicates significant) are shown for the entire sample and for each group of TF and TS. **(A)** Swing-leg thigh angle at contralateral touchdown. **(B)** Swing-leg knee angle at contralateral touchdown.

**Figure 5:**
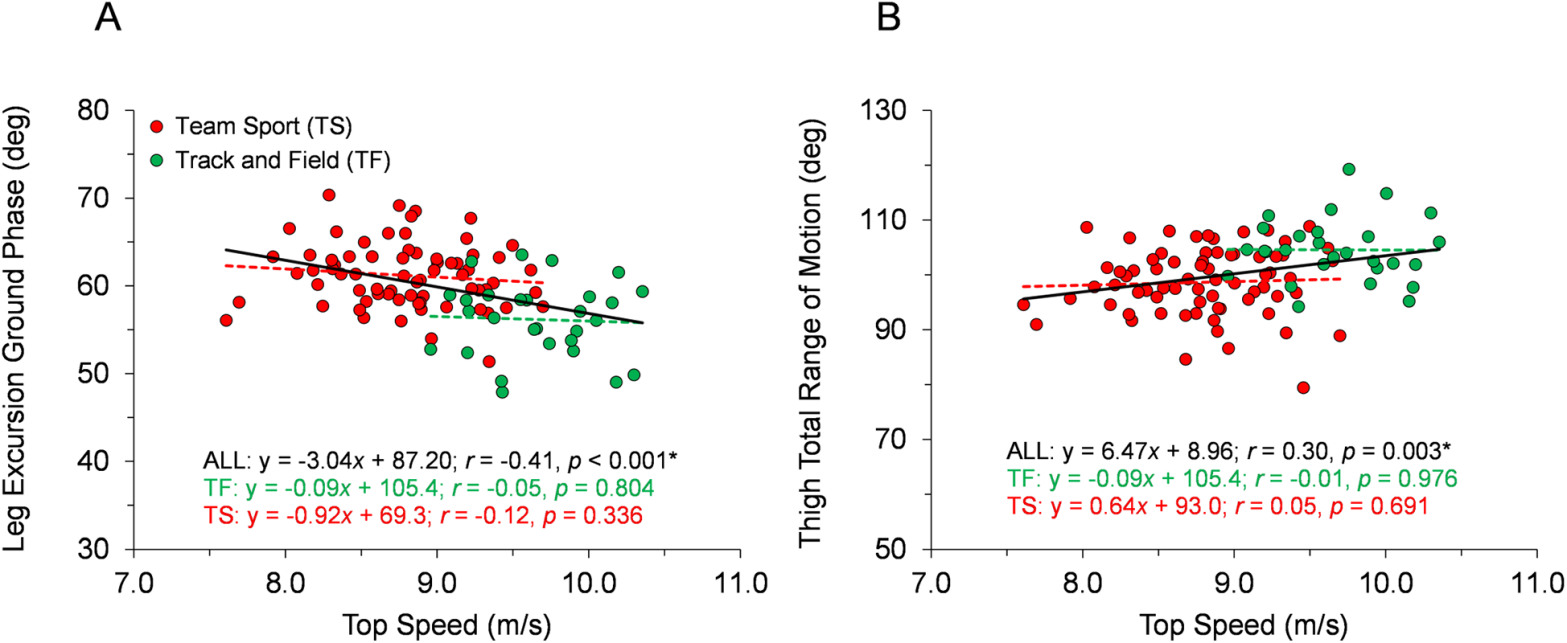
Angular position data across the range of top speeds. Trend lines with best-fit linear regression equations, correlation coefficients (Pearson's *r* or Spearman's *ρ*), and *p*-values (* indicates significant) are shown for the entire sample and for each group of TF and TS. **(A)** Leg excursion angle during ground phase from touchdown to takeoff. **(B)** Thigh total range of motion during the swing phase.

**Figure 6:**
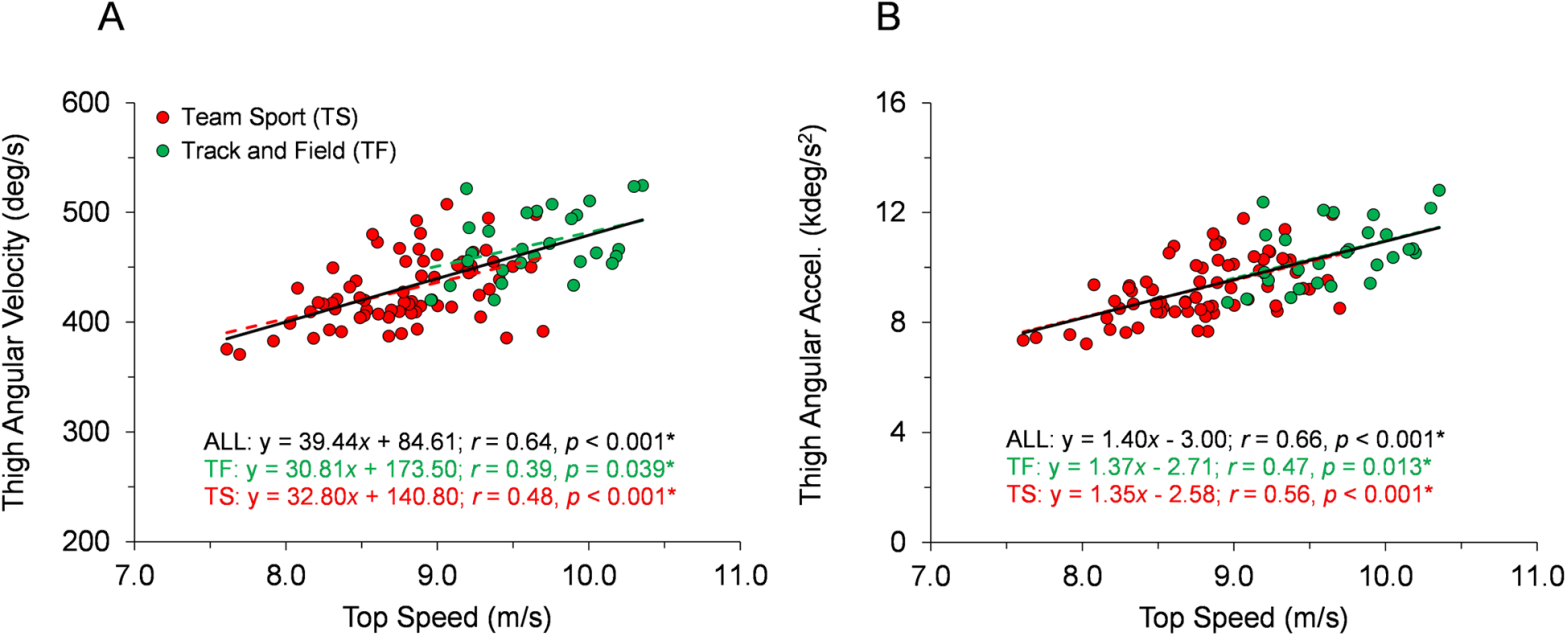
Angular rate data across the range of top speeds. Trend lines with best-fit linear regression equations, correlation coefficients (Pearson's *r* or Spearman's *ρ*), and *p*-values (* indicates significant) are shown for the entire sample and for each group of TF and TS. **(A)** Average thigh angular velocity during the entire gait cycle. **(B)** Peak thigh angular acceleration during the entire gait cycle.

With respect to the first hypothesis, Figures 2–6 display data for each key kinematic variable across the range of top speeds. These figures include the correlation coefficients, *p*-values, and best-fit linear regression equations for the entire sample and each group of TF and TS. Additionally, to facilitate interpretation of the data, Table 1 presents mean ± SD for each variable with the participants categorized into the five sub-groups of Fast TF, Slow TF, Fast TS, Intermediate TS, and Slow TS. As displayed in Figures 2–6, when analyzed across the entire sample, each key kinematic variable was significantly related to top speed, with correlations ranging from 0.30 ≤ |*r* or *ρ*| ≤ 0.66. When analyzed within the TF and TS groups, both average thigh angular velocity and peak thigh angular acceleration were significantly related to top speed. However, none of the key angular position variables were significantly correlated with top speed when analyzed within the TF group, and only three of the eight key angular position variables were significantly correlated with top speed when analyzed within the TS group (COM-foot angle at touchdown, foot angle at touchdown, and swing-leg knee angle at contralateral touchdown).

**Table 1:** Descriptive statistics for participants divided into five sub-groups based on athletic background (track and field [TF] or team sport [TS]) and top speed: fast TF, slow TF, fast TS, intermediate TS, and slow TS. All values are listed as mean ± standard deviation (SD).

Measurement	Fast TF	Slow TF	Fast TS	Intermediate TS	Slow TS
	(*n* = 14)	(*n* = 14)	(*n* = 22)	(*n* = 29)	(*n* = 19)
Height (m)	1.79 ± 0.05	1.78 ± 0.06	1.82 ± 0.07	1.81 ± 0.07	1.81 ± 0.05
Body Mass (kg)	75.76 ± 5.73	74.50 ± 6.68	81.72 ± 8.30	82.45 ± 7.53	87.51 ± 9.59
Top Speed (m/s)	10.00 ± 0.21	9.34 ± 0.21	9.31 ± 0.19	8.78 ± 0.14	8.21 ± 0.25
Maximum Thigh Extension during Swing (deg)	−28.44 ± 6.76	−28.98 ± 5.90	−34.17 ± 4.66	−35.46 ± 4.59	−33.70 ± 4.18
Maximum Thigh Flexion during Swing (deg)	76.16 ± 5.89	75.54 ± 6.64	64.93 ± 6.79	63.04 ± 4.62	64.61 ± 4.83
COM-Foot Leg Angle at Touchdown (deg)	12.71 ± 2.38	14.19 ± 2.09	16.11 ± 1.84	17.04 ± 1.81	17.85 ± 2.11
Foot Angle at Touchdown (deg)	8.56 ± 4.84	5.40 ± 4.49	0.24 ± 6.49	0.15 ± 5.46	−5.69 ± 7.50
Swing-Leg Thigh Angle at Contralateral TD (deg)	15.43 ± 13.72	10.83 ± 15.55	−7.24 ± 9.98	−10.68 ± 7.11	−9.86 ± 8.40
Swing-Leg Knee Angle at Contralateral TD (deg)	49.30 ± 6.95	52.57 ± 8.16	61.73 ± 6.95	68.18 ± 7.06	66.36 ± 5.83
Leg Excursion Angle Touchdown to Takeoff (deg)	55.88 ± 4.06	56.43 ± 4.59	60.62 ± 3.54	61.21 ± 3.92	61.84 ± 3.46
Thigh Total Range of Motion (deg)	104.60 ± 6.67	104.52 ± 4.87	99.11 ± 7.11	98.50 ± 6.31	98.30 ± 4.72
Average Thigh Angular Velocity (deg/s)	482.91 ± 28.91	460.22 ± 29.56	444.92 ± 31.56	430.49 ± 30.18	408.69 ± 21.77
Peak Thigh Angular Acceleration (kdeg/s^2^)	11.02 ± 0.92	10.03 ± 1.18	9.88 ± 1.01	9.32 ± 1.06	8.40 ± 0.77

**Figure 7:**
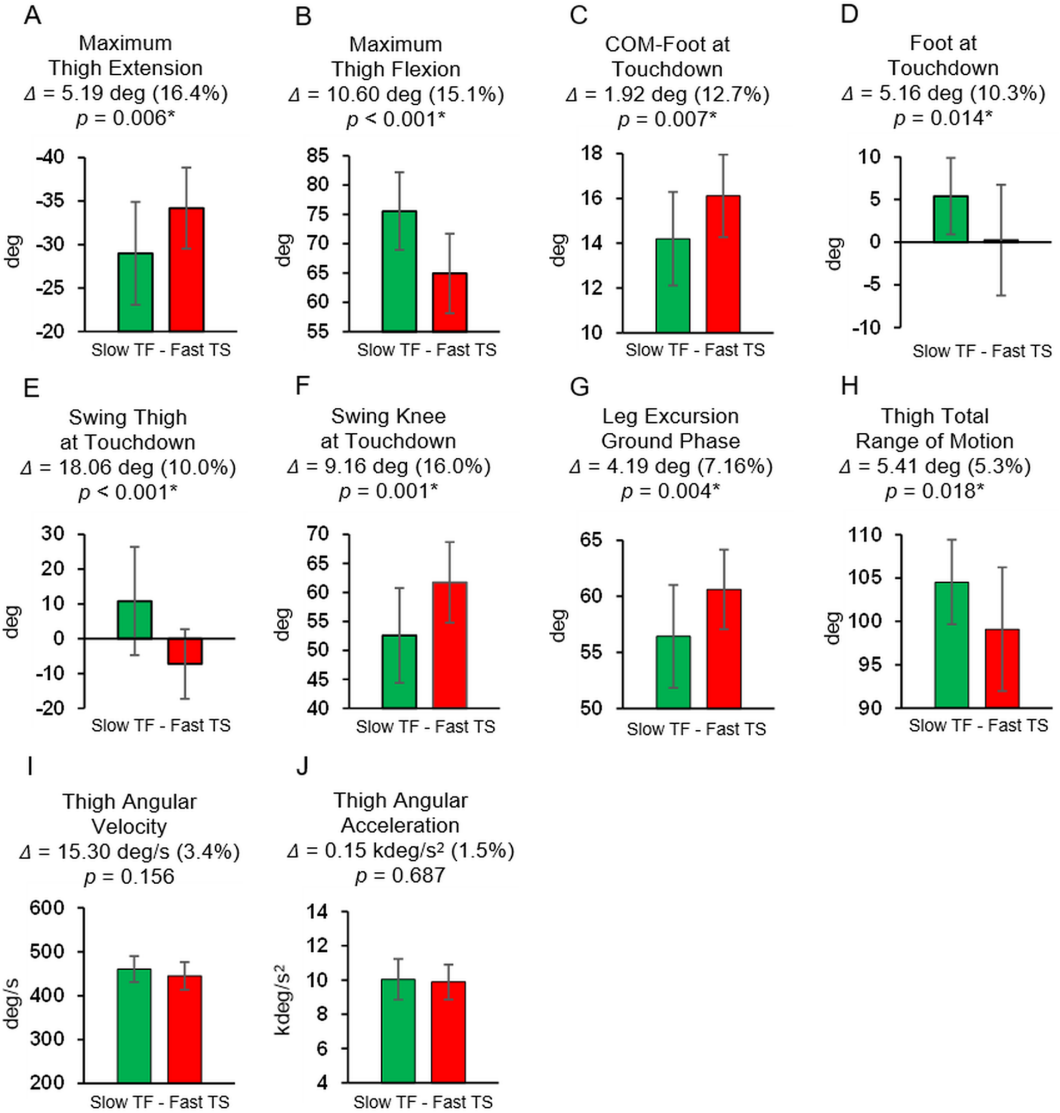
Results of the independent *t*-tests comparing slow track and field (slow TF, green) versus fast team sport (fast TS, red) for the angular kinematic variables. Absolute differences (Δ), percentage differences (%), and *p*-values (* indicates significant) are listed. No significant differences in top sprinting speed existed between these sub-groups (Slow TF: 9.34 ± 0.21 m/s; Fast TS: 9.31 ± 0.19 m/s; Δ = 0.03 m/s [0.3%], *p* = 0.636). **(A)** Maximum thigh extension angle. **(B)** Maximum thigh flexion angle. **(C)** COM-Foot angle at touchdown. **(D)** Foot angle at touchdown. **(E)** Swing thigh angle at touchdown. **(F)** Swing knee angle at touchdown. **(G)** Leg excursion angle during the ground phase. **(H)** Thigh total range of motion. **(I)** Thigh angular velocity. **(J)** Thigh angular acceleration.

With respect to the second hypothesis, independent *t*-tests confirmed that no significant differences in top sprinting speed existed between the sub-groups of Slow TF and Fast TS (Slow TF: 9.34 ± 0.21 m/s; Fast TS: 9.31 ± 0.19 m/s; Δ = 0.03 m/s [0.3%], *p* = 0.636). However, as presented in Figure 7, significant differences did exist between Slow TF and Fast TS for all other measures except average thigh angular velocity and peak thigh angular acceleration (Figures 7I,J). Fast TS athletes achieved similar top speed compared to Slow TF but did so with increased thigh extension and decreased thigh flexion during the swing phase (Figures 7A,B), a greater COM-foot angle at touchdown and a more flat-footed ground contact at touchdown (Figures 7C,D), a more posterior position of the swing-leg thigh and more extended swing-leg knee at contralateral touchdown (Figures 7E,F), and a larger leg excursion angle during the ground contact phase and decreased thigh total range of motion during the swing phase (Figures 7G,H). Absolute and percentage differences between Slow TF and Fast TS for each of these key kinematic variables, and the accompanying *p*-values from the independent *t*-tests, are listed in Figure 7.

**Figure 8:**
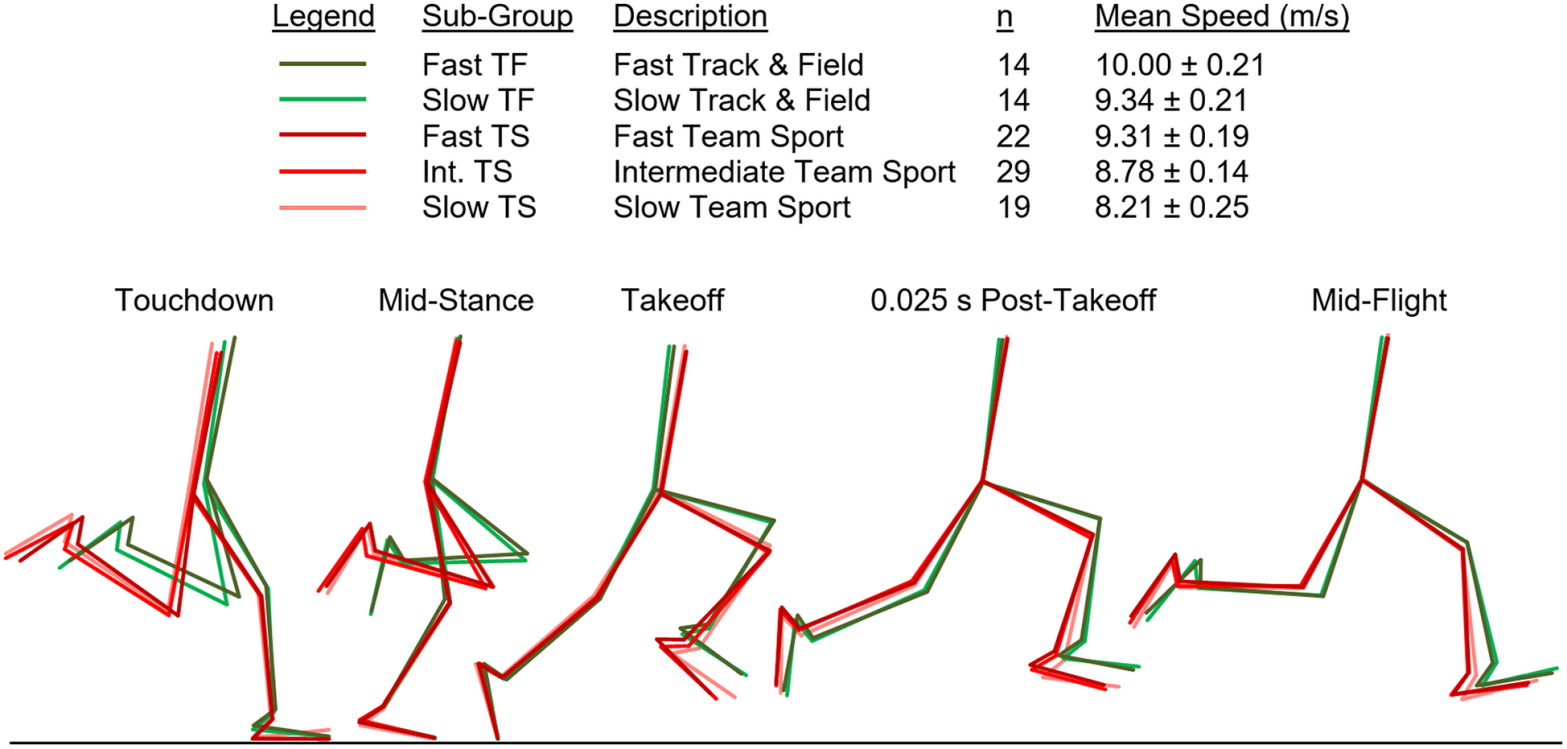
Computer-generated composite stick figures for all sub-groups. Stick figures during ground contact were aligned to the most anterior position of the foot while on the running surface. Stick figures during flight were aligned at the hip joint. All angular position values for the seven-segment models at each event frame are available in the Supplementary Materials. Slow TF (9.34 ± 0.21 m/s) and Fast TS (9.31 ± 0.19 m/s) had no significant difference in top speed.

Additionally, for illustrative purposes, group-mean composite stick figures for all five sub-groups are displayed in Figure 8. The figure was computer-generated using the digitized data to create the seven-segment models for each sub-group and to align them at each event. The stick figures during ground contact (touchdown, mid-stance, and takeoff) were aligned to the most anterior position of the foot while on the running surface. The stick figures during flight (post-takeoff and mid-flight) were aligned at the hip. Segment angles for all five sub-groups and the correlational analyses for all joint angles at the five event frames are available in the Supplementary Materials.

## Discussion

### Summary of findings

In this study we investigated angular kinematics during top speed sprinting in male intercollegiate TF and TS athletes. Based on prior experimental research and applied coaching experience, our first hypothesis was that key kinematic variables would each be correlated with top speed both across the entire sample of participants and within groups of TF and TS athletes. Our second hypothesis was that when examining sub-groups of TF and TS athletes of similar top speeds, different angular positional strategies would be utilized to attain similar top speeds.

Our first hypothesis was partially supported by the results, as each key kinematic variable was significantly related to top speed when analyzed across the entire sample. However, counter to expectations, the only variables that were correlated with top speed when analyzed within both TF and TS groups were average thigh angular velocity and peak thigh angular acceleration. Only three out of the eight key angular position variables were significantly correlated with top speed when analyzed within the TS group and none of the key angular position variables were correlated with top speed in the TF group. As displayed in Figures 2–6 and Table 1, there were appreciable between-group differences for TF vs. TS when analyzing the variables but not large differences within TF or TS groups for most of the key angular position variables. There were discrete differences in angular positions between the TF and TS groups but strikingly similar angular positions within their respective TF or TS groups (Figure 8). It can be observed that the continuum of angular values across the entire heterogenous sample is a composition of two distinct angular position strategies (see trendlines in Figures 2–5).

The results fully supported the second hypothesis, as both Slow TF and Fast TS sub-groups had mean top speeds of ∼9.3 m/s, and yet the key angular position variables demonstrated significant differences between these two sub-groups. Fast TS athletes achieved similar top speed as Slow TF athletes but did so with a less front-side and more ground-based positional strategy. Specifically, this included increased thigh extension and decreased thigh flexion during the swing phase, larger leg excursion angle during the ground contact phase, and smaller thigh total range of motion during the swing phase (Figures 7A,B,G,H, 8, Table 1). Additionally, there were significant differences between Slow TF and Fast TS in positions of both the stance- and swing-leg at touchdown, with Fast TS exhibiting a larger COM-foot angle at touchdown and a more flat-footed initial ground contact, a more posterior position of the swing-leg thigh, and a more extended swing-leg knee at contralateral touchdown (Figures 7C–F, 8, Table 1).

### Comparison to prior research

The data collected in the present investigation generally agree with findings from prior research examining angular kinematic variables in a variety of athletic populations. In the present study, faster runners displayed more front-side mechanics when analyzed across the entire sample, although similar to Haugen et al. (2), this trend was not apparent when examined within homogeneous groupings of athletes (Figure 2 and Table 1). Similarly, data for thigh total range of motion was positively correlated with top speed both across the entire sample in the present study and in prior research examining a heterogeneous sample (7). Although in this study and others (26), this variable was not correlated with top speed in homogeneous groups of runners (Figure 5B). Finally, data related to thigh angular velocity and thigh angular acceleration have been significantly correlated with top speed in heterogeneous samples both in the present study (Figure 6) and in prior research (7, 13). However, while still significantly correlated when analyzed within TF and TS athletes, the associations were not as consistent in homogeneous groups both in this study and other studies (26). Generally speaking, our data provide further evidence that certain kinematic variables are significantly related to top speed in heterogeneous samples, but also that statistical correlations may become less prominent when examined in groups of runners with similar athletic backgrounds.

Moreover, the results from this study provide further context to recent findings highlighting the different kinematic strategies employed by TS athletes compared to TF athletes, even when running at similar top speeds. Specifically, Meng et al. (18) reported that TS athletes clearly demonstrate a more “ground-based” strategy compared to their TF counterparts, including longer ground contact times and contact lengths, shorter flight times and flight lengths, and increased duty factors. In the present study, the increased leg excursion angle during the ground contact phase and decreased thigh total range of motion during swing exhibited by TS athletes (Figures 5, 7G,H, Table 1) directly align with the ground-based strategy for TS described by Meng et al. (18). Furthermore, the significantly larger COM-foot angle at touchdown and more flat-footed initial ground contact position displayed here by TS athletes (Figures 7C,D, Table 1) would be related to prolonged ground contact times and contact lengths, as reported in Meng et al. (18).

### Practical applications

Clearly, the aforementioned results illustrate different kinematic strategies for TF and TS of similar top speed. This could be explained by the agility demands of team-sport game play (change of direction in response to an opponent) and less technique-focused top speed training in TS athletes. The Fast TS and Slow TF exhibited substantially different angular positions (Figure 8, Table 1, and Supplementary Materials), while attaining nearly identical mean top speeds (9.34 vs. 9.31 m/s). Conversely, the Fast TF and Slow TF sub-groups exhibited nearly identical angular positions (Figure 8, Table 1, and Supplementary Materials), while attaining substantially different mean top speeds (10.00 vs. 9.34 m/s). These findings prompt two interesting questions regarding the determinants of top sprinting speed. What is the role of technique? What is the role of physical capacity?

In contrast to the key angular position variables (Figures 2–5), the key angular rate variables (average thigh angular velocity and peak thigh angular acceleration, Figure 6) were significantly related to top speed across the entire sample of participants and within both groups of TF and TS athletes. Furthermore, there were no significant between-group differences in thigh angular velocity or thigh angular acceleration for Slow TF vs. Fast TS (Figures 7I,J), indicating that similar values for these two variables could be achieved even when the angular positions were different between Slow TF and Fast TS sub-groups. Therefore, as it relates to top speed performance and angular kinematics, the variables relating to the physical capacity to rotate the thighs were consistently correlated with top speed, whereas these correlations were not consistently significant across TF and TS groups for the key angular position variables.

Of course, running mechanics are modifiable, and recent evidence suggests that angular kinematics can shift towards a more front-side technical model after a period of targeted sprint training (14). Limb coordination is also related to top speed (27) and training-induced improvements in sprinting performance may correspond to changes in lower extremity technique and coordination (14, 28). Furthermore, the posture and angular positions that an athlete displays while sprinting are important for reasons other than top speed, as sprinting technique may be specifically linked to soft tissue injuries such as hamstring strain (29). In fact, many of the variables associated with better sprint performance, such as increased limb angular velocities and accelerations during swing, and increased ground reaction forces during stance, can actually present a greater challenge for the hamstring muscles (30). Therefore, even though most of the key angular position variables in this study were not correlated with top speed when analyzed within a homogeneous group of TF or TS athletes, aiming to modify running mechanics in order to align with positions consistent with reduced risk of soft tissue injury (29, 31, 32) is still a logical focal point for sprint training.

### Limitations and future research

This research study analyzed lower extremity angular variables, with data collected in a field-based setting on a track using a single camera video-based protocol. Thus, kinematic data for both near- and far-side body segments were determined using a two-dimensional manual digitizing routine instead of a three-dimensional motion capture system in a laboratory setting. This approach was utilized since the motion occurred primarily along the sagittal plane and it allowed for effective testing of a large number of athletes in a single session, with intra-rater reliability metrics that demonstrated replicable results. The development of the experimental protocol and accompanying analysis procedures described in this investigation provide a straightforward method for researchers and practitioners to collect and analyze data when working with athletes in the field.

The participants of this study were male intercollegiate athletes, including TF athletes who competed primarily in the sprint events, and TS athletes from the sports of lacrosse, soccer, and baseball. Future research should investigate if the limb segment and angular positions observed in the present study are also exhibited by elite- or professional-level TF and TS athletes, in TS athletes from different sporting backgrounds such as American football or the rugby codes, and for athletes of different body dimensions and anthropometrics. Correspondingly similar studies are needed for female athletes from TF sprint events and from TS competition at both the intercollegiate and elite levels. Extended investigations examining the changes in these variables after a period of training may provide additional insights. It is plausible that a longitudinal modification to the angular position variables could enable an increase in the key angular rate variables (or vice versa), resulting in an improvement in top speed.

Therefore, the continued development of normative data and technical models specific to TS athletes is imperative. The ground-based kinematic strategies displayed by Fast TS in the present investigation, including longer leg excursion angles during the ground contact phase and lower position of the front limb during the swing phase could be beneficial. This may be related to the constraints and demands of team sport competition, including reactive multi-directional maneuvers, repeated sprints, and holding a ball or implement. Consequently, the traditional front-side technical model often utilized with TF athletes may need modification to be more applicable to TS athletes.

## Summary and conclusion

In this study we investigated the top speed sprinting mechanics in intercollegiate male TF and TS athletes. Our first hypothesis was partially supported, as each key kinematic variable was significantly correlated with top speed when analyzed across the entire heterogeneous sample, but most key angular position variables were not correlated when analyzed within groups of TF or TS athletes. This was due to the distinct positional strategy adopted by each TF and TS group with only minimal variations of the strategy within each group. Our second hypothesis was fully supported, as Slow TF and Fast TS athletes of similar top speeds demonstrated substantially different angular positions, indicating that Fast TS athletes typically sprint with a less front-side and more ground-based strategy compared to their Slow TF counterparts. For the athletes in this investigation, the physical capacity to rotate the limbs (thigh angular velocity and acceleration) was correlated with top speed both across the entire sample of participants and within groups of TF and TS athletes. Future research should focus on continuing to develop technical models for sprinting that are focused on TS athletes and exploring the effects of training interventions on technique and performance during top speed sprinting. Supplementary Materials.

## Data Availability

The original contributions presented in the study are included in the article/Supplementary Material, further inquiries can be directed to the corresponding author.
