# Supplementary material for: Angular kinematics during top speed sprinting in male intercollegiate track and field and team sport athletes
Source: Front Sports Act Living. 2025 Mar 31;7:1535798. doi: 10.3389/fspor.2025.1535798 (PMC11994691; doi:10.3389/fspor.2025.1535798)
Supplement: Supplementary file 1 [file Datasheet1.pdf]

Supplementary Materials
Angular Position Data and Kinetics Data

Angular Kinematics during Top Speed Sprinting in Male Intercollegiate Track and Field and Team Sport Athletes

*Kenneth P. Clark[1], Christopher R. Meng[1,2], Cory T. Walts[3],
Laurence J. Ryan[4], and David J. Stearne[1]

[1]Department of Kinesiology, West Chester University, West Chester, PA USA
[2]Department of Athletics, Princeton University, Princeton NJ USA
[3]Department of Athletics, University of Pennsylvania, Philadelphia PA USA
[4]Independent Researcher, Dallas TX USA

Angular Position Data

Supplementary materials in this section include angular position data to accompany the Clark et al. manuscript "Angular Kinematics during Top Speed Sprinting in Male Intercollegiate Track and Field and Team Sport Athletes."

The angular position data include the following:

Figure S1 - Kinematic definitions for the angular position data.

Table S1 - Sub-group data.

Table S2 - Seven-segment model angles for the five sub-groups at each of the five event frames displayed in Figure 8 of the main manuscript.

Table S3 - Group data.

Table S4 - Correlational analyses for the lower extremity joint angles at each of the five event frames for the Entire Sample (n = 98), TF Only (n = 28), and TS Only (n = 70).

Table S5 - Correlation coefficients with confidence intervals for the key kinematic variables in Figures 2-6 in the main manuscript.

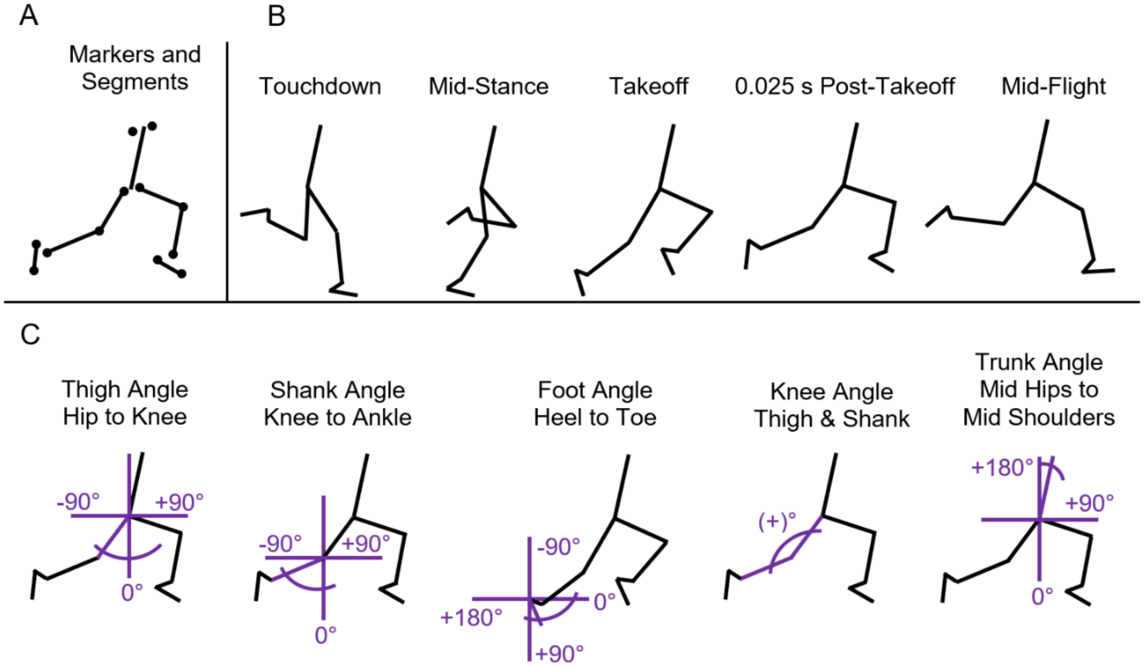

Figure S1. Kinematic Definitions. (A) Twelve marker locations were digitized at the toes and heels of the shoes, and the joint centers of rotation of the ankles, knees, hips, and shoulders to create a seven-segment model. **(B)** Five event frames for each step were digitized. **(C)** Angular position conventions for the segment angles.

Table S1 - Sub-group data. Values are Mean ± Standard Deviation SD.

Sub-Group	Description	Number	Height (m)	Mass (kg)	Top Speed Category	Mean Speed (m/s)
Fast TF	Fast Track & Field	n = 14	1.79 ± 0.05	75.76 ± 5.73	Top Speed > 9.65 m/s	10.00 ± 0.21
Slow TF	Slow Track & Field	n = 14	1.78 ± 0.06	74.50 ± 6.68	Top Speed < 9.65 m/s	9.34 ± 0.21
Fast TS	Fast Team Sport	n = 22	1.82 ± 0.07	81.72 ± 8.30	Top Speed > 9.0 m/s	9.31 ± 0.19
Int. TS	Intermediate Team Sport	n = 29	1.81 ± 0.07	82.45 ± 7.53	Top Speed = 8.5-9.00 m/s	8.78 ± 0.14
Slow TS	Slow Team Sport	n = 19	1.81 ± 0.05	87.51 ± 9.59	Top Speed < 8.5 m/s	8.21 ± 0.25

Table S2 – Model angles. Seven-segment model angles for the five sub-groups at each of the five event frames displayed in Figure 8 of the main manuscript. Values are Mean Degrees ± Standard Deviation SD.

		Touchdown		Mid-Stance		Takeoff		0.025 s Post-Takeoff		Mid-Flight	
Angle	Sub-Group	Mean	± SD	Mean	± SD	Mean	± SD	Mean	± SD	Mean	± SD
Stance Leg Thigh	Fast TF	29.10	4.35	6.52	4.67	-26.70	5.21	-27.06	7.86	-18.31	9.55
	Slow TF	31.66	6.55	8.36	4.42	-27.00	4.55	-28.55	6.46	-19.44	9.59
	Fast TS	33.53	3.80	10.61	3.02	-31.19	3.38	-33.71	4.89	-28.67	6.58
	Int. TS	34.08	3.08	11.48	2.73	-31.97	3.45	-35.08	4.90	-30.00	4.85
	Slow TS	34.29	3.62	11.10	2.02	-32.04	3.17	-33.03	4.68	-28.31	6.14
Stance Leg Shank	Fast TF	4.43	3.34	-29.74	4.63	-48.99	3.05	-67.72	3.82	-94.14	5.06
	Slow TF	2.79	2.89	-32.49	5.58	-49.09	3.23	-65.46	3.70	-93.39	4.46
	Fast TS	5.04	2.50	-33.22	3.56	-50.21	2.19	-68.17	2.74	-92.83	3.65
	Int. TS	5.54	2.45	-32.73	3.26	-49.29	3.31	-66.51	3.96	-89.83	2.60
	Slow TS	5.76	2.80	-33.01	3.04	-49.24	3.38	-66.44	3.04	-90.21	3.08
Stance Leg Foot	Fast TF	8.56	4.84	12.50	1.99	79.39	4.51	100.69	4.80	135.68	9.95
	Slow TF	5.40	4.49	12.99	3.28	76.77	5.82	96.14	3.84	128.37	9.14
	Fast TS	0.24	6.49	11.84	3.21	75.20	5.22	94.22	5.87	125.36	8.06
	Int. TS	0.15	5.46	13.37	2.85	75.84	3.57	92.96	3.83	123.33	5.98
	Slow TS	-5.69	7.50	10.03	2.28	73.65	4.81	90.55	4.51	120.90	6.32
Stance Leg Knee	Fast TF	155.33	6.51	143.73	8.14	157.71	6.49	139.34	9.04	104.18	9.15
	Slow TF	151.13	8.92	139.15	9.66	157.91	5.86	143.08	7.67	106.05	9.83
	Fast TS	151.51	4.84	136.17	5.82	160.98	4.44	145.54	6.48	115.84	6.24
	Int. TS	151.46	3.57	135.79	4.90	162.68	5.28	148.57	7.81	120.17	4.85
	Slow TS	151.47	4.78	135.89	4.05	162.79	6.04	146.58	6.98	118.10	5.67
Swing Leg Thigh	Fast TF	15.43	13.72	51.70	11.32	75.23	6.35	72.94	4.45	59.22	4.12
	Slow TF	10.83	15.55	49.35	14.59	74.41	7.64	72.65	5.23	59.22	5.06
	Fast TS	-7.24	9.98	32.22	10.35	62.48	8.22	64.02	6.26	55.49	5.48
	Int. TS	-10.68	7.11	29.35	8.07	61.15	5.41	62.14	4.33	54.99	3.70
	Slow TS	-9.86	8.40	32.95	10.70	63.51	5.52	62.81	4.23	54.54	3.39
Swing Leg Shank	Fast TF	-115.27	13.85	-86.61	15.81	-32.43	11.32	-8.72	9.10	11.96	7.22
	Slow TF	-116.60	13.11	-88.62	22.18	-30.40	18.13	-7.14	15.18	14.00	10.38
	Fast TS	-125.51	10.61	-107.13	15.09	-43.77	13.78	-18.22	11.01	2.91	6.84
	Int. TS	-122.50	8.22	-105.40	11.53	-40.71	10.43	-16.65	9.14	2.60	6.40
	Slow TS	-123.50	8.19	-104.87	15.49	-34.90	12.67	-12.40	9.65	6.29	6.25
Swing Leg Foot	Fast TF	145.33	21.66	104.24	23.43	36.96	15.94	10.94	13.61	-9.51	9.40
	Slow TF	143.09	17.27	102.82	25.75	32.81	20.57	6.22	16.21	-13.28	9.16
	Fast TS	144.77	35.17	124.68	18.78	45.34	15.29	15.13	12.32	-8.94	6.72
	Int. TS	152.23	11.59	125.10	16.74	43.48	13.60	14.96	10.99	-7.32	7.89
	Slow TS	149.80	9.78	121.27	19.81	34.40	13.69	6.94	11.19	-13.98	7.26
Swing Leg Knee	Fast TF	49.30	6.95	41.69	7.20	72.34	7.52	98.34	8.52	132.74	9.49
	Slow TF	52.57	8.16	42.03	10.17	75.19	13.35	100.21	14.41	134.78	13.19
	Fast TS	61.73	6.95	40.65	8.05	73.75	10.73	97.76	11.52	127.42	10.62
	Int. TS	68.18	7.06	45.25	6.12	78.15	7.71	101.21	8.95	127.61	7.13
	Slow TS	66.36	5.83	42.18	6.65	81.60	9.52	104.79	9.88	131.76	7.06
Trunk	Fast TF	168.68	2.55	168.12	2.98	172.43	1.87	172.21	2.00	170.15	2.86
	Slow TF	171.68	3.88	169.93	2.59	173.65	2.92	173.59	2.90	172.00	3.03
	Fast TS	169.02	3.70	166.88	3.57	169.92	3.96	169.99	3.96	169.44	3.99
	Int. TS	169.94	3.66	167.48	3.92	170.03	4.26	170.18	4.49	169.57	4.14
	Slow TS	170.74	3.40	168.17	3.21	170.03	3.09	170.06	2.88	169.67	3.09

Table S3 - Group data. Values are Mean ± Standard Deviation SD.

Group	Description	Number	Height (m)	Mass (kg)
Entire Sample	All Participants	n = 98	1.80 ± 0.06	81.17 ± 8.82
TF Only	Track & Field	n = 28	1.79 ± 0.05	75.13 ± 6.14
TS Only	Team Sport	n = 70	1.81 ± 0.07	83.59 ± 8.59

Table S4 - Group correlational analyses. Group correlational analyses for the lower extremity joint angles. Pearson's "r" is listed for normally distributed data, and Spearman's "ρ" is listed for nonnormally distributed data. Statistically significant values at $p < 0.05$ are signified with an *.

Angle	Group	Touchdown	Mid-Stance	Takeoff	0.025 s Post-Takeoff	Mid-Flight
Stance Leg Thigh	Entire Sample	**r = -0.33***	**ρ = -0.30***	**ρ = 0.36***	**ρ = 0.30***	**ρ = 0.37***
	TF Only	r = -0.23	r = -0.22	r = 0.07	r = 0.16	r = 0.11
	TS Only	r = -0.07	r = -0.09	r = -0.10	r = -0.01	r = -0.01
Stance Leg Shank	Entire Sample	**r = -0.21***	r = 0.19	r = -0.06	r = -0.19	**ρ = -0.041***
	TF Only	r = 0.31	r = 0.34	r = -0.07	r = -0.37	r = -0.20
	TS Only	r = -0.17	r = -0.08	r = -0.19	**r = -0.28***	**r = -0.33***
Stance Leg Foot	Entire Sample	**r = 0.56***	**r = 0.20***	**r = 0.28***	**r = 0.58***	**ρ = 0.46***
	TF Only	r = 0.30	r = -0.09	r = 0.16	**ρ = 0.58***	**r = 0.42***
	TS Only	**r = 0.35***	**r = 0.24***	r = 0.12	**r = 0.34***	**r = 0.26***
Stance Leg Knee	Entire Sample	r = 0.15	**ρ = 0.24***	**r = -0.33***	**r = -0.34***	**ρ = -0.54***
	TF Only	r = 0.29	r = 0.30	r = -0.10	r = -0.30	r = -0.21
	TS Only	r = -0.04	r = -0.01	r = -0.17	r = -0.12	r = -0.18
Swing Leg Thigh	Entire Sample	**ρ = 0.55***	**ρ = 0.44***	**ρ = 0.42***	**ρ = 0.51***	**ρ = 0.37***
	TF Only	r = 0.26	r = 0.20	r = 0.12	r = 0.02	r = -0.10
	TS Only	r = 0.16	r = -0.01	r = -0.05	r = 0.12	r = 0.11
Swing Leg Shank	Entire Sample	ρ = 0.13	**ρ = 0.30***	r = 0.07	r = 0.14	**ρ = 0.26***
	TF Only	ρ = 0.13	r = 0.20	r = 0.08	r = 0.09	r = 0.10
	TS Only	r = -0.08	r = -0.04	**r = -0.27***	r = -0.20	ρ = -0.17
Swing Leg Foot	Entire Sample	ρ = -0.06	ρ = -0.25	r = 0.02	r = 0.03	r = 0.06
	TF Only	r = 0.01	r = -0.06	r = 0.02	r = 0.05	r = 0.06
	TS Only	ρ = 0.01	r = 0.03	**r = 0.28***	**r = 0.24***	r = 0.21
Swing Leg Knee	Entire Sample	**r = -0.60***	r = -0.05	**r = -0.28***	r = 0.10	ρ = 0.09
	TF Only	r = -0.24	r = 0.14	r = 0.03	r = 0.09	r = 0.12
	TS Only	**r = -0.29***	r = -0.06	**r = -0.32***	**r = -0.25***	r = -0.19

Table S5 – Correlation coefficients and confidence intervals. Correlation coefficients with 95% confidence intervals for the key kinematic variables in Figures 2-6 in the main manuscript.

All Participants (ALL)

Variable	Correlation Coefficient	95% Confidence Interval		
Maximum Thigh Extension	0.33	0.14	to	0.50
Maximum Thigh Flexion	0.50	0.33	to	0.64
COM-Foot Angle at Touchdown	-0.61	-0.72	to	-0.46
Foot Angle at Touchdown	0.56	0.41	to	0.68
Swing Thigh Angle at Touchdown	0.55	0.39	to	0.68
Swing Knee Angle at Touchdown	-0.61	-0.72	to	-0.46
Leg Excursion Angle Ground Phase	-0.41	-0.56	to	-0.23
Thigh Total Range of Motion	0.30	0.11	to	0.47
Thigh Angular Velocity	0.64	0.51	to	0.75
Thigh Angular Acceleration	0.66	0.53	to	0.76

Track & Field (TF)

Variable	Correlation Coefficient	95% Confidence Interval		
Maximum Thigh Extension	0.11	-0.28	to	0.46
Maximum Thigh Flexion	0.10	-0.28	to	0.46
COM-Foot Angle at Touchdown	-0.31	-0.61	to	0.07
Foot Angle at Touchdown	0.29	-0.09	to	0.60
Swing Thigh Angle at Touchdown	0.25	-0.13	to	0.57
Swing Knee Angle at Touchdown	-0.24	-0.57	to	0.14
Leg Excursion Angle Ground Phase	-0.05	-0.41	to	0.33
Thigh Total Range of Motion	-0.01	-0.38	to	0.37
Thigh Angular Velocity	0.39	0.02	to	0.67
Thigh Angular Acceleration	0.47	0.11	to	0.71

Team Sports (TS)

Variable	Correlation Coefficient	95% Confidence Interval		
Maximum Thigh Extension	-0.01	-0.24	to	0.23
Maximum Thigh Flexion	0.05	-0.19	to	0.28
COM-Foot Angle at Touchdown	-0.35	-0.54	to	-0.12
Foot Angle at Touchdown	0.35	0.12	to	0.54
Swing Thigh Angle at Touchdown	0.16	-0.08	to	0.38
Swing Knee Angle at Touchdown	-0.29	-0.49	to	-0.06
Leg Excursion Angle Ground Phase	-0.12	-0.34	to	0.12
Thigh Total Range of Motion	0.05	-0.19	to	0.28
Thigh Angular Velocity	0.48	0.28	to	0.64
Thigh Angular Acceleration	0.56	0.37	to	0.70

Kinetics Data

Supplementary materials in this section include kinetics data to accompany the Clark et al. manuscript "Angular Kinematics during Top Speed Sprinting in Male Intercollegiate Track and Field and Team Sport Athletes."

Values of vertical force (F_{vert}), vertical stiffness (k_{vert}), and leg stiffness (k_{leg}) were determined from this data set using the spatiotemporal values presented in Meng et al. (2024). Average vertical force normalized to body weight was determined using the methods of Weyand et al. (2000) from their Equation 2. Vertical stiffness and leg stiffness were determined using the methods of Morin et al. (2005) from their equations in the Appendix. Maximum force and displacements were normalized to body weight and leg length following the procedures of Hof (1996) resulting in dimensionless values for vertical stiffness and leg stiffness.

Correlational analysis was performed to determine the relationship between top speed and vertical force, vertical stiffness, and leg stiffness. For each variable, the normality of data was checked using the Shapiro-Wilk test. Normally distributed data were analyzed with Pearson's r and non-normally distributed data were analyzed with Spearman's ρ. For graphic purposes, linear regression was also performed to generate a best-fit equation with variable x representing top speed. The correlational analyses and linear regression were completed across the Entire Sample (n = 98) and separately for TF Only (n = 28) and TS Only (n = 70).

Separate independent t-tests were conducted for each variable to specifically examine significant differences between the sub-groups of Slow TF and Fast TS athletes. The normality of data was checked using the Shapiro-Wilk test. Normally distributed data were analyzed with parametric independent t-tests and non-normally distributed data were analyzed with the Mann-Whitney test.

The kinetics data include:

Table S6 - Sub-group data.

Table S7 - Kinetics data for vertical force, vertical stiffness, and leg stiffness for each sub-group.

Figure S2 - Kinetics data plots and correlational analyses across the range of top speeds.

Figure S3 - Results of the independent t-tests comparing Slow Track and Field versus Fast Team Sport for the kinetic variables.

Figure S2 shows that each kinetic variable was significantly related to top speed when analyzed across the entire sample. The trend lines clearly indicate the discrete differences between the TF and TS groups. Figure S3 shows these distinct levels between the Slow TF and Fast TS sub-groups. The ground-based strategy, as indicated by the spatiotemporal analyses from Meng et al. (2024) and the angular kinematics analyses from this study, shows that Fast TS attained the same top speed as Slow TF but with lower measures of vertical force, vertical stiffness, and leg stiffness.

References:

Hof, A. L. (1996). Scaling gait data to body size. *Gait & Posture*, 3 (4), 222-223.

Meng, C. R., Walts, C. T., Ryan, L. J., Stearne, D. J., & Clark, K. P. (2024). Spatiotemporal kinematics during top speed sprinting in male intercollegiate track and field and team sport athletes. *Sports Biomechanics*, 1-14

Morin, J. B., Dalleau, G., Kyröläinen, H., Jeannin, T., & Belli, A. (2005). A simple method for measuring stiffness during running. *Journal of applied biomechanics*, 21(2), 167-180.

Weyand, P. G., Sternlight, D. B., Bellizzi, M. J., & Wright, S. (2000). Faster top running speeds are achieved with greater ground forces not more rapid leg movements. *Journal of Applied Physiology*, 89 (5), 1991-1999.

Table S6 - Sub-group data. Values are Mean ± Standard Deviation SD.

Sub-Group	Description	Number	Height (m)	Mass (kg)	Top Speed Category	Mean Speed (m/s)
Fast TF	Fast Track & Field	n = 14	1.79 ± 0.05	75.76 ± 5.73	Top Speed > 9.65 m/s	10.00 ± 0.21
Slow TF	Slow Track & Field	n = 14	1.78 ± 0.06	74.50 ± 6.68	Top Speed < 9.65 m/s	9.34 ± 0.21
Fast TS	Fast Team Sport	n = 22	1.82 ± 0.07	81.72 ± 8.30	Top Speed > 9.0 m/s	9.31 ± 0.19
Int. TS	Intermediate Team Sport	n = 29	1.81 ± 0.07	82.45 ± 7.53	Top Speed = 8.5-9.00 m/s	8.78 ± 0.14
Slow TS	Slow Team Sport	n = 19	1.81 ± 0.05	87.51 ± 9.59	Top Speed < 8.5 m/s	8.21 ± 0.25

Table S7 - Kinetics data. Kinetics data for vertical force, vertical stiffness, and leg stiffness for each subgroup. Forces were normalized to body weight, displacements were normalized to leg length, resulting in dimensionless values for force and stiffness. Values are Mean ± Standard Deviation SD.

Kinetic Variable	Fast TF	Slow TF	Fast TS	Int. TS	Slow TS
Normalized Average Vertical Force (F_{vert})	2.25 ± 0.18	2.21 ± 0.21	1.97 ± 0.13	1.90 ± 0.09	1.86 ± 0.09
Normalized Vertical Stiffness (k_{vert})	159.92 ± 21.15	140.29 ± 19.95	126.90 ± 15.25	113.58 ± 12.54	100.18 ± 9.32
Normalized Leg Stiffness (k_{leg})	31.44 ± 8.59	31.20 ± 9.89	22.65 ± 4.67	21.43 ± 3.77	21.33 ± 3.70

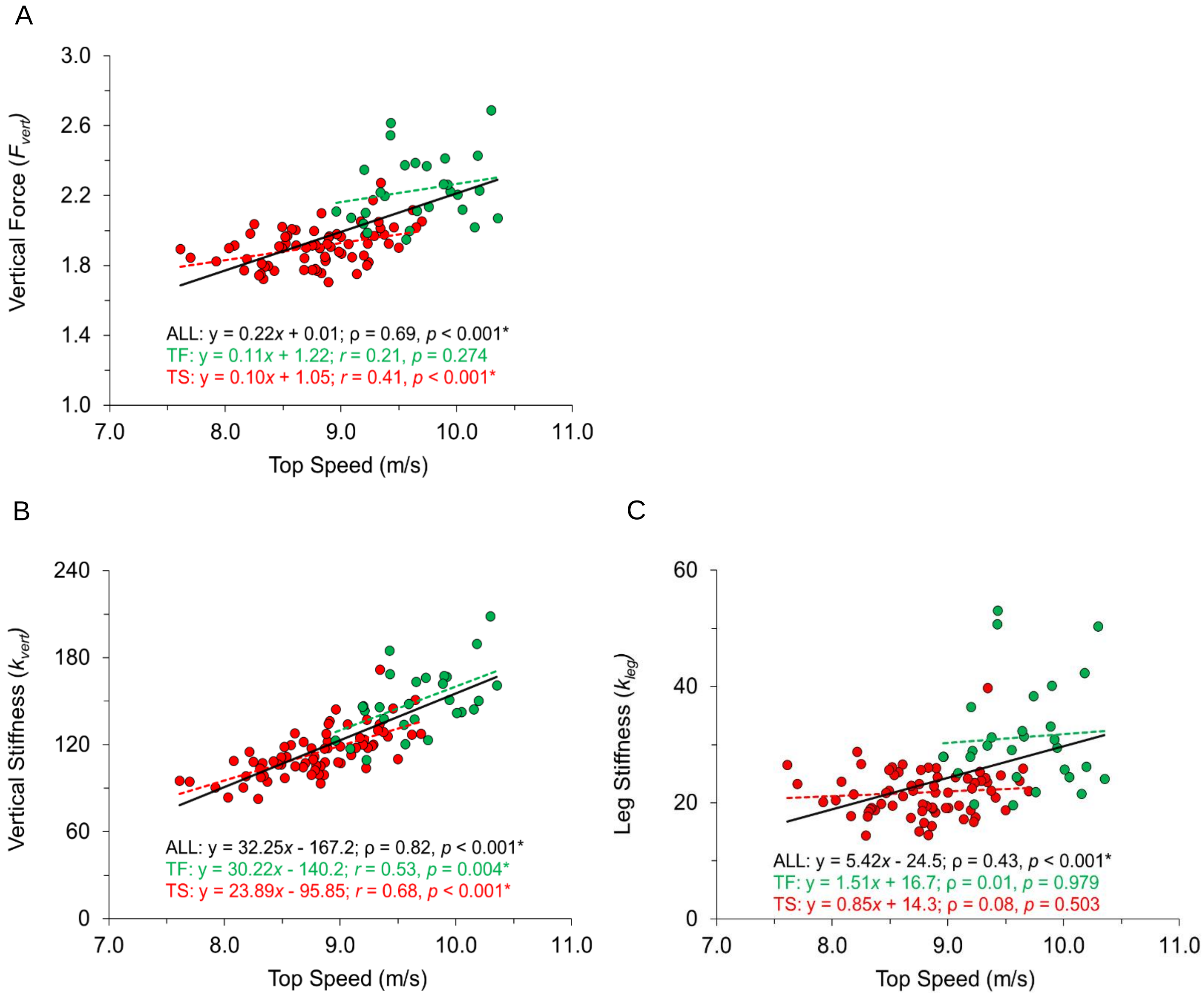

Figure S2. Kinetics data. Kinetics data plots across the range of top speeds. Trend lines with best-fit linear regression equations, correlation coefficients (Pearson's r or Spearman's ρ), and p-values (* indicates significant) are shown for the entire sample and for each group of TF and TS. **(A)** Normalized average vertical force. **(B)** Normalized vertical stiffness. **(C)** Normalized leg stiffness.

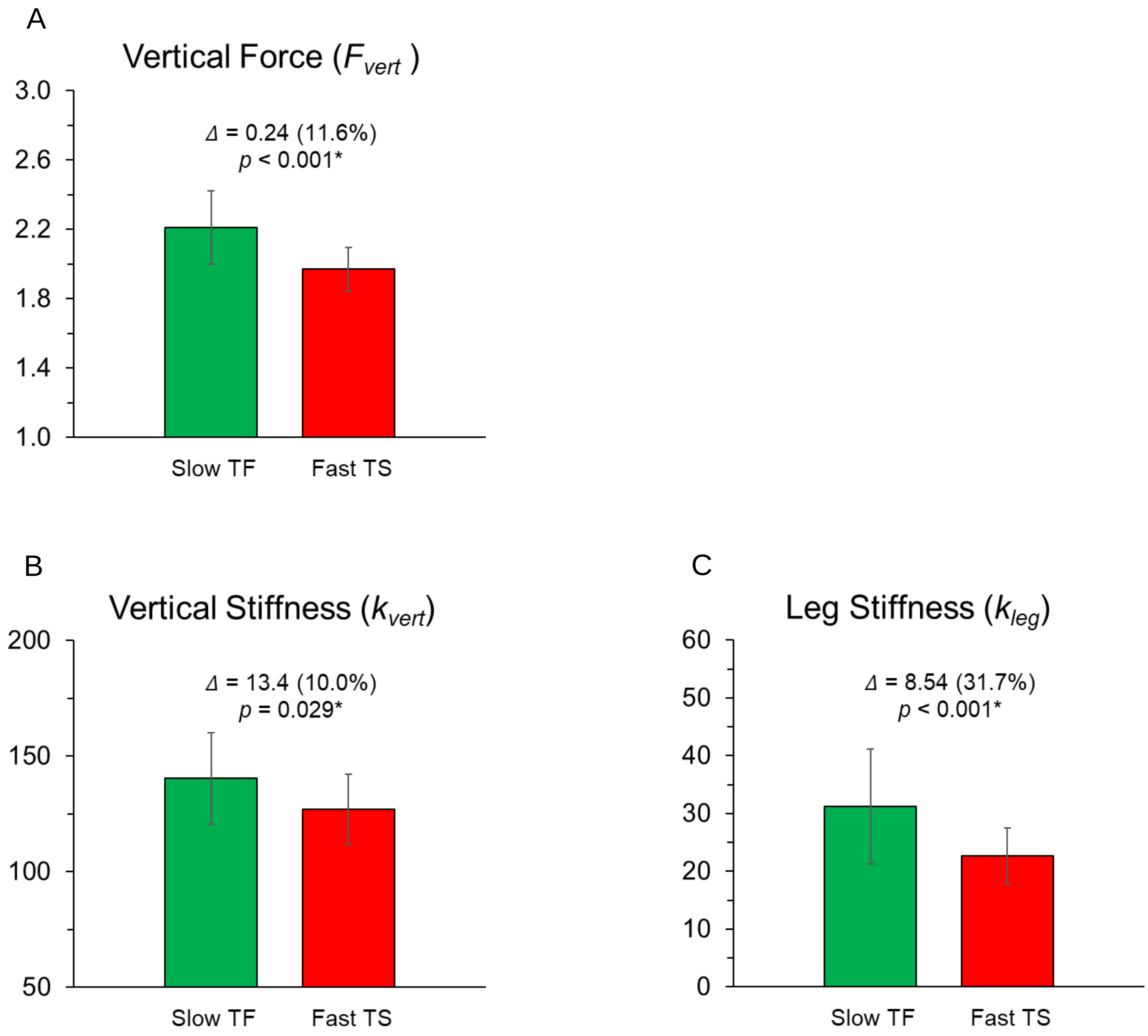

Figure S3. Independent t-tests. Results of the independent t-tests comparing Slow Track and Field (Slow TF, green) versus Fast Team Sport (Fast TS, red) for the kinetic variables. Absolute differences (Δ), percentage differences (%), and *p*-values (* indicates significant) are listed. No significant differences in top sprinting speed existed between these sub-groups. **(A)** Normalized average vertical force. **(B)** Normalized vertical stiffness. **(C)** Normalized leg stiffness.
